# Multiplex PCR for the simultaneous detection of the Enterobacterial gene *wecA*, the Shiga Toxin genes (*stx*_1_ and *stx*_2_) and the Intimin gene (*eae*)

**DOI:** 10.1186/s13104-018-3457-8

**Published:** 2018-06-07

**Authors:** Marc B. Anglès d’Auriac, Reidun Sirevåg

**Affiliations:** 1 Norwegian Institute for Water Research (NIVA), 0349 Oslo, Norway; 2 Department of Biosciences, University of Oslo, Box 1031, Blindern, 0316 Oslo, Norway

**Keywords:** *eae*, Enterobacteriaceae, Enterobacterial Common Antigen (ECA), Multiplex PCR, *stx*, Diagnostic, Faecal indicator bacteria (FIB), Indicator organisms

## Abstract

**Objectives:**

The aetiology of several human diarrhoeas has been increasingly associated with the presence of virulence factors rather than with the bacterial species hosting the virulence genes, exemplified by the sporadic emergence of new bacterial hosts. Two important virulence factors are the Shiga toxin (Stx) and the *E. coli* outer membrane protein (Eae) or intimin, encoded by the *stx* and *eae* genes, respectively. Although several polymerase chain reaction (PCR) protocols target these virulence genes, few aim at detecting all variants or have an internal amplification control (IAC) included in a multiplex assay. The objective of this work was to develop a simple multiplex PCR assay in order to detect all *stx* and *eae* variants, as well as to detect bacteria belonging to the Enterobacteriaceae, also used as an IAC.

**Results:**

The *wecA* gene coding for the production of the Enterobacterial Common Antigen was used to develop an Enterobacteriaceae specific qPCR. Universal primers for the detection of *stx* and *eae* were developed and linked to a wecA primer pair in a robust triplex PCR. In addition, subtyping of the *stx* genes was achieved by subjecting the PCR products to restriction digestion and semi-nested duplex PCR, providing a simple screening assay for human diarrhoea diagnostic.

**Electronic supplementary material:**

The online version of this article (10.1186/s13104-018-3457-8) contains supplementary material, which is available to authorized users.

## Introduction

According to WHO diarrhoeal disease is the second cause of death of children less than 5 years old, killing around half a million every year [1]. Hence, easy to implement diagnostic tests are highly relevant for patient care as well as for food and water monitoring. Once associated only with specific bacterial strains or serotypes, some diseases have been caused by new strains that were not previously identified as food/water-borne pathogens. A major mechanism associated with the emergence of pathogens is horizontal gene transfer enabling the sudden acquisition of virulence factors [2, 3] also between phylogenetically distant bacterial genera [4]. Among the numerous known virulence genes, *stx* and *eae* play a major role in the virulence of various enteric pathogens members of the Enterobacteriaceae [5–7]. Detecting the presence of these virulence genes is now a common procedure for tracking the associated aetiologic agents although other markers have also recently been used to monitor diarrhoeic *E. coli* [8]. The Shiga toxin producing *E. coli* (STEC) comprises the enterohaemorrhagic *E. coli* (EHEC) pathotype and is defined by the presence of the *stx* gene. Two main types of this virulence factor have been defined, stx_1_ (quasi identical to Stx produced by *Shigella dysenteriae*) and stx_2_, both of which are further divided in several subtypes [9]. Most STEC detection methods rely on the identification of serotype O157H7. As more than 50% of STEC infections may be caused by non-O157H7 strains, the need for diagnostic methods detecting all types of STEC has been emphasized [10, 11]. The gene *eae* coding for intimin has been found in enteropathogenic *E. coli* (EPEC), EHEC, *E. albertii* and *Citrobacter rodentium* [12].

Several multiplex polymerase chain reaction (PCR) methods for the detection of virulence factors and serogroup markers associated with human gastrointestinal diseases have been previously developed which include the presence of an internal amplification control (IAC) [13–16], or of an indicator organism such as *E. coli* to provide a positive control [17–21] (see Additional file 1). *E. coli* belongs to the Enterobacteriaceae, a group which includes the total coliforms (TC) and most enteric pathogens and is described as a “general (process) microbial indicator” [22] used for assessing the efficiency of food and water treatment. Therefore, in the present method, the Enterobacteriaceae was chosen to serve both as a general microbial indicator of sample contamination, and as a positive control for the multiplex PCR when tracking the presence of *stx* and *eae* genes. The “universal” primer design for the detection of *stx* and *eae* virulence genetic elements aims at detecting all variants of the gene regardless of the bacterial host species whereas other “universal” stx and eae primers, aim at *E. coli* (Additional file 1). In addition, sequence variability within the *stx* PCR product was exploited in order to develop a nested PCR and a restriction digestion protocol for typing of the genes. To our knowledge it is the first time such screening “universal” *stx* and *eae* primers are used in a multiplex PCR with an Enterobacteriaceae IAC.

## Main Text

### Materials and methods

#### Bacterial strains and culture media

For testing the multiplex protocol, the 23 bacterial isolates used are listed in Additional file 2. The bacterial strains were provided by The Norwegian National Institute for Health (Folkehelsa) and the Rikshospitalet University Hospital. Pure cultures were grown in 10 ml Luria Broth over night at 37 °C with shaking. Grown cultures were boiled for 10 min, and serially diluted in water before being used for PCR analysis. Alternatively, single colonies were picked from agar plates, suspended in water and used directly for the PCR.

#### Software and primers

Sequence alignments were performed using the Multalin web site http://multalin.toulouse.inra.fr/multalin/ accessed 1 February 2018 [23]. Primer design as well as restriction enzyme analyses were performed with Oligo 6.0 (Molecular Biology Insights, Inc., USA) software or performed manually using the alignments results and guidelines [24–27]. Amplicon product sizes and primer sequence are shown in Table 1.

**Table 1:** Primer sequences, product sizes and conditions used in the triplex and simplex PCRs

Primer sets	Target genes	Primers			MgCl_2_ (mM)	Product size (bp)
		Names	Sequences (5′–3′)^a^	μM		
A	*wecA*	Meca202U	GGGTTGTCCTGCGTCTCGTT	0.05	3	452
		Meca633L	TATTCTGCCAGCACGCCAATG			
	*stx*_1_ and *stx*_2_	UstxU1	TRTTGARCRAAATAATTTATATGT	0.5		526 (*stx*_1_)
		UstxL1	MTGATGATGRCAATTCAGTAT			523 (*stx*_2_)
	*stxA*_2f_	UstxU3	AATGGAACGGAATAACTTATATGT	0.05		523
		UstxL3	GGTTGAGTGGCAATTAAGGAT			
	*eae* intimin	Ueae28U	ACCCGGCACAAGCATAAG	0.1		741
		Ueae748L	CGTAAAGCGRGAGTCAATRTA			
B	*stx*_1_ and *stx*_2_	UstxL1	MTGATGATGRCAATTCAGTAT	0.1	3	
	*stx*_1_	Nestx1	GTACAACACTKGATGATCTC	0.3		200
	*stx*_2_	Nestx2	TGACRACGGACAGCAGT	0.05		410
C	*wecA*	Meca480UU21	GGATATGGTGGCGATTATGTA	0.2	2	226
		Meca685LU21	GAATGCTAGCAAAAAGAGCAC			
		Meca479UU21	TGGATATGGTGGCGATTATGT	0.2	2	261
		Meca722LU18	TCCAGGCMCGCTTAATGC			

Triplex PCR (A) semi-nested duplex PCR for stx_1_ and stx_2_ typing (B) and simplex PCR for Enterobacteriaceae (C)

^a^R = A or G, M = A or C, K = G or T

#### PCR amplification

Samples (10 μl) were amplified in 50 μl final reaction mixtures using a BioTest Biometra or a TGRADIENT (Whatman-Biometra^R^) PCR thermocycler. For the semi-nested duplex PCR, a 1000-fold dilution of the triplex reaction was used. The buffered (1×) mixtures contained 0.1 mM nucleotides and 0.2 U of DyNazyme II (Finnzymes) DNA polymerase. The concentration of primers and MgCl_2_ for the triplex PCR and the semi-nested duplex PCR were as indicated in Table 1. For the simplex stx PCR, 0.1 μM of primers UstxU1 and UstxL1 and 0.01 μM UstxU3 and UstxL3 were used. Thermocycling conditions were as follows: 2 min preheating at 94 °C followed by 40 (triplex and simplex), or 25 (semi-nested duplex PCR) cycles of 94 °C for 15 s, 57 °C for 30 s and 72 °C for 60 s. PCR products were separated by electrophoresis on a 1.7% 0.5 × Tris–borate–EDTA agarose gel stained with ethidium bromide, visualized using 75 V and 25 mA for 1 h 30 min and then photographed under UV illumination.

#### Restriction endonuclease

The reaction mixture contained 16 μl PCR products, 10 U *Bsr*I restriction endonuclease (New England Biolab) with the provided NEB3 buffer in a total volume of 20 μl. Digestion was performed in PCR tubes at 65 °C for 2 h 30 min in the thermocycler after which 10 μl of the sample was analysed by electrophoresis as described for PCR amplification.

## Results

### Virulence factors

A total of 45 different *stxA* gene sequences and 21 different *eaeA* gene sequences were aligned (see A1 and A2, respectively in [28]). The primers were aimed at detecting all variants of *stx* and *eae*, and thus were designed on the basis of the most conserved area of the DNA sequence alignment. An alignment of the universal degenerate Ustx primers with the most relevant primer pairs used in other PCR protocols is shown in Additional file 3.

### Triplex PCR

**Fig. 1:**
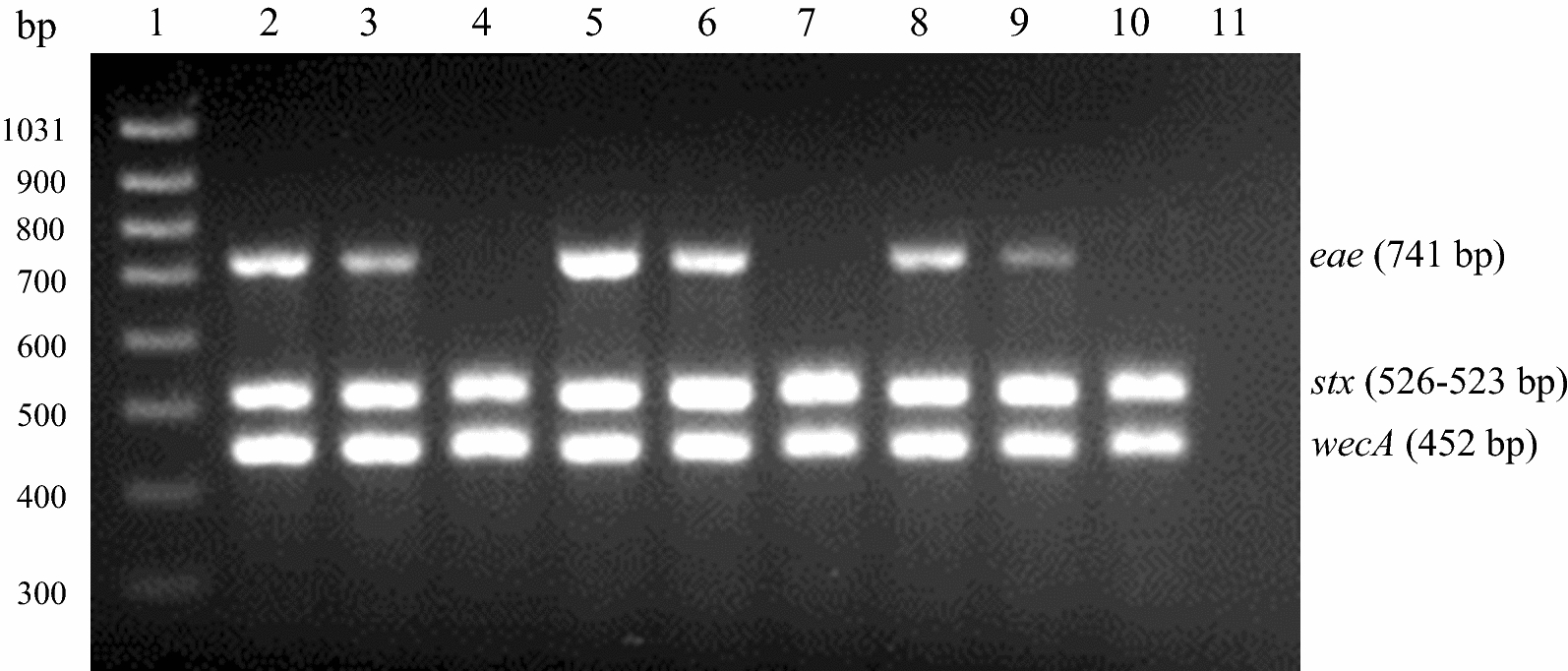
Triplex PCR optimization for MgCl_2_ concentration. *Lanes 2*–*4* 2 mM MgCl_2_, *Lanes 5*–*7* 3 mM MgCl_2_, *Lanes 8*–*10* 4 mM MgCl_2_, *Lane 1* DNA size ladder, *Lanes 2*, *5* and *8 E. coli* O157:H7, *Lanes 3*, *6* and *9 E. coli* O157:H7 and *S. dysenteriae*, *Lanes 4*, *7* and *10 S. dysenteriae*, *Lane 11* negative control

**Fig. 2:**
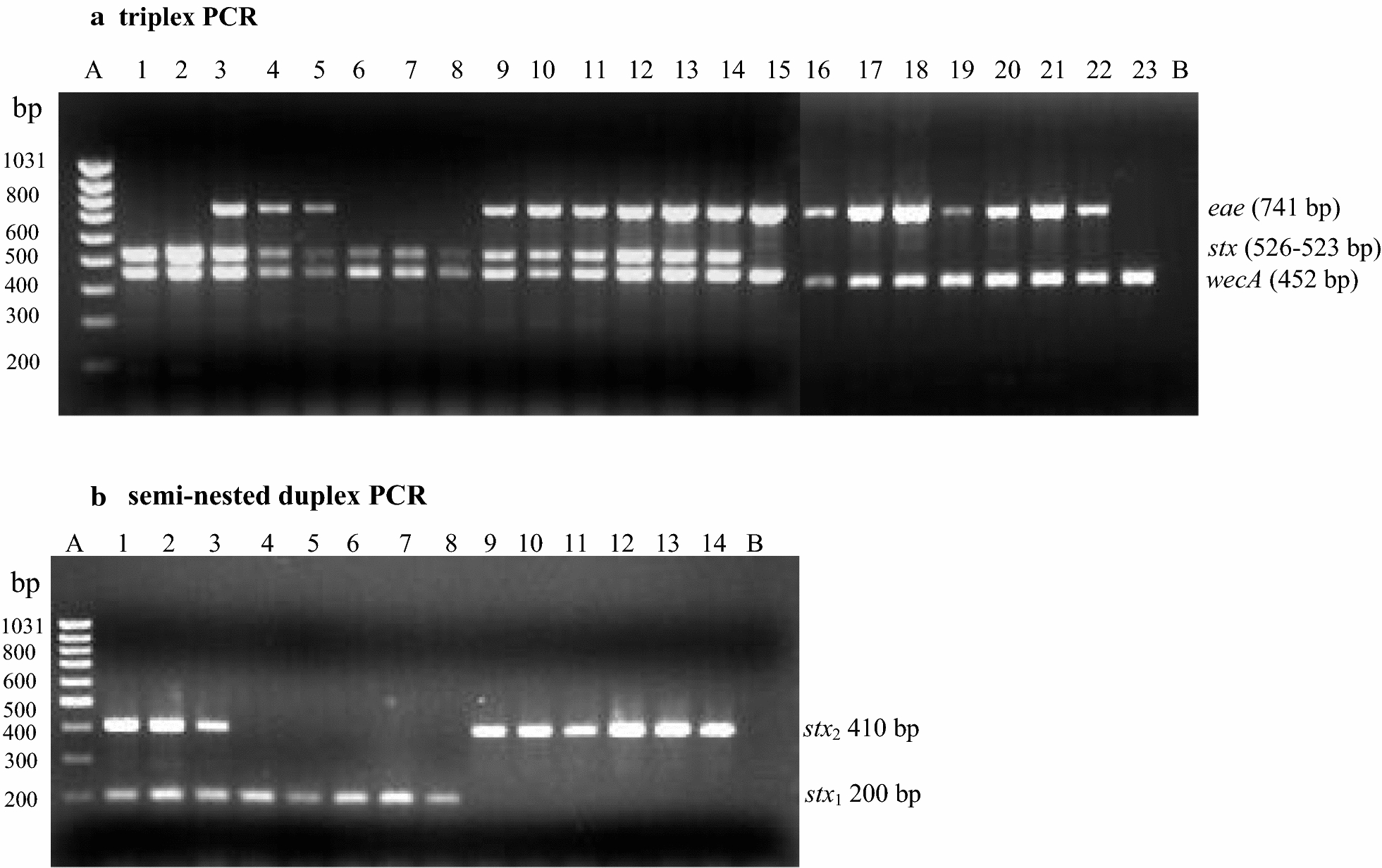
Multiplex PCR products gel electrophoresis results. **a** Triplex PCR performed on 23 bacterial strains of *E. coli* and *S. dysenteriae* showing the amplicon products for *eae*, *stx* and *wecA* genes. *Lane A* DNA size ladder, *Lanes 1–23* bacteria as listed in Additional file 2, *Lane B* negative control. **b** Semi-nested duplex PCR using as template the *stx* universal PCR amplicon (526–523 bp). *Lane A* DNA size ladder, *Lanes 1–14* stx positive strains as listed in Additional file 2 and shown in **a** *Lanes 1–14*, *Lane B* negative control

The triplex PCR was developed to simultaneously detect Enterobacteriaceae, used as an IAC, and the presence or absence of any variants of *stx* and *eae* genes. The triplex PCR was optimized varying annealing temperature, primer concentration and by testing additives or facilitators such as DMSO, glycerol, bovine serum albumin, formamide and MgCl_2_ which are reported to improve multiplex PCR [24, 29, 30]. Only increasing the concentration of MgCl_2_ from 2 to 3 mM improved results as shown in Fig. 1. The results from testing 19 pathogenic *E. coli*, 3 *S. dysenteriae* and 1 non-pathogenic *E. coli*, for specificity of the assay are shown in Fig. 2a and Additional file 2.

### Semi-nested duplex PCR for differentiating *stx*_1_ and *stx*_2_

The semi-nested duplex PCR consisted of UstxL1, used as the reverse primer in the triplex PCR, and two new forward primers, Nestx1 and Nestx2. These two forward primers are complementary to *stx*_1_ and *stx*_2_ respectively and are located within the amplicon produced in the triplex. The products of the amplification consist of 200 bp and 410 bp for *stx*_1_ and *stx*_2_, respectively. The assay was tested on 14 strains containing *stx*_1_ or *stx*_2_, or both, and the results are shown in Fig. 2b. These results were corroborated by those produced by the restriction enzymatic digestion assay shown in Additional files 4 and 5.

### Enzymatic restriction digestion for typing

The result obtained after digestion with *Bsr*I of the *stx* simplex PCR product is shown in Additional file 4. The fragment pattern enables the distinction of four different groups of stx variants, *stx*_1c_, *stx*_1_, *stx*_2_ + *stx*_2c_ + *stx*_2d_, and *stx*_2e_ + *stx*_2f_ as shown in Additional file 5. The smallest fragment, of 39 bp, could not be visualised on the agarose gel, but this does not affect the interpretation of the results. Similarly, the 62 bp fragment produced from *stx*_1_ was not always visible. However, this fragment was not required for positive identification of *stx*_1_, which was specifically identified by the presence of the 334 bp fragment.

## Discussion

### Enterobacterial Common Antigen

The Enterobacterial Common Antigen (ECA) was first described in 1963 by Kunin [31] and is defined as a cross-reactive antigen that is detectable in all genera of Enterobacteriaceae by several methods including using antisera to *E. coli* [32]. ECA was later found to be strictly family specific with diagnostic potential because of its universal presence in the family (see reviews [32]). Two of the genes implicated in the ECA synthesis are the *rfe* and *rff* genes [33, 34] later renamed *wec* [35]. Immunology-based diagnostic tests have been developed to detect the presence of ECA for clinical applications [36] and later to monitor the quality of drinking water by probing for bacteria belonging to the Enterobacteriaceae family [37]. In the PCR based protocols used here, the *wecA* primers detected all tested 23 strains of *E. coli* and *S. dysenteriae*.

### Virulence genes

Eighteen varieties of intimin have been described [38] among which: α (alpha), β (beta), γ (gamma), δ (delta) [39] and ε (epsilon) [40]. The *eae* gene is found in the locus of enterocyte effacement (LEE) pathogenicity island of both EPEC and EHEC [41, 42]. In the present study, all 6 tested EPEC were *eae*-positive as well as 11 EHEC out of 13 tested. Of the two negatives, EHEC O113:H21 has also been reported *eae*-negative in a previous study [43].

Among the two *stx* groups, the second, *stx*_2_ and its five variants, is the most diverse and includes the most potent Shiga toxins for humans. Both *stx*_2_ and *stx*_2c_ are mainly hosted by STEC associated with the aetiology of severe human diarrhoea whereas *stx*_2d_ has been isolated in STEC from human as well as cattle origin [44]. Finally, *stx*_2e_ is found in porcine STEC [45] while *stx*_2f_ is found in STEC hosted by birds [46, 47]. Although the toxins Stx_2e_ and Stx_2f_ seem to be adapted to their respective hosts, both have been associated with human disease [45, 48]. Swine was also shown to harbour STEC carrying *stx*_1_ and *stx*_2d_ associated with human infections [49], a finding which further underlines the importance of establishing screening methods designed for detecting all variants. The results obtained for *stx* in the present study were in agreement with expectations. All three *S. dysenteriae* were positive for *stx* by triplex PCR and further confirmed to harbour *stx*_1_ by both semi-nested duplex PCR and enzymatic restriction. Among the 13 EHEC strains, 11 were found *stx* positive by triplex PCR. Strain ATCC 43888 was *stx*-negative as expected whereas strain 3005/00 was unexpectedly *stx*-negative although *eae*-positive. This could be the result of loss of the virulence factor as it has been previously demonstrated for stx both in vivo [50] and in vitro [51].

Three EHEC strains were shown to have both *stx*_1_ and *stx*_2_, confirmed by both the semi-nested duplex PCR and the enzymatic restriction typing method. In particular, strain BE97-2317 was shown by enzymatic restriction to harbour *stx*_1c_, a gene coding for a toxin type also previously found associated with EHEC O128:H2 and negative for *eae* [43].

Various universal primer pairs for the detection of *stx* have been described in [52–59] some which overlap with the Ustx primers employed in this study (Additional file 3). However, few primers are able to detect all variants and are used in a multiplex assay [60–62] or also have integrated an IAC such as *E. coli* detection [19]. Integrating the detection of an indicator group, expected to be co-detected along with the targeted virulence markers, has the advantages over using a traditional IAC that it will also be able to detect, not only PCR inhibition or failure, but also absence of DNA. Finally, the Enterobacteriaceae family has been described as a possible alternative to faecal indicator bacteria, as it can better reflect the hygienic status of food products [63], hence Enterobacteriaceae PCR assays should find several areas of applications. Overall, this simple molecular screening assay including its further typing possibility for *stx*, should help food and health authorities to increase their monitoring efforts to improve water and food microbiological quality as well as patient diagnostic capabilities.

## Limitations

A limited number of strains were used for detection capability of the assay, and specificity testing for Enterobacteriaceae was not performed. Limit of detection has not been evaluated.

## Additional files

**Additional file 1.** Overview of qPCR protocols for detection of *stx*, *eae*, other *E. coli * pathogenic markers, serotyping genes and indicator markers.

**Additional file 2.** List of strains with triplex PCR results.

**Additional file 3.** Comparison of selected primers aiming at the detection of all variants of *stxA* gene; arrows are indicating the direction of the primers and complementary sequences are shown for the reverse primers. The conserved bases are shaded in black while the variable positions are unshaded.

**Additional file 4.** PCR and BsrI digestion. Lane A, DNA size ladder; Lane 1 to 28, *stx* positive strains as listed in Additional file 2, odd numbers are the undigested *stx* products, even numbers are the digestion products.

**Additional file 5.** Subtyping results for *stx* by restriction enzymatic digestion of the *stx* amplicon and semi-nested duplex PCR.

## Abbreviations

Eae: *E. coli* attaching and effacing
ECA: Enterobacterial Common Antigen
EHEC: Enterohaemorrhagic *E. coli*
EPEC: Enteropathogenic *E. coli*
PCR: Polymerase chain reaction
IAC: Internal amplification control
STEC: Shiga toxin producing *E. coli*
Stx: Shiga toxin
TC: Total coliforms
