# Supplementary material for: Multiplex PCR for the simultaneous detection of the Enterobacterial gene wecA, the Shiga Toxin genes (stx1 and stx2) and the Intimin gene (eae)
Source: BMC Res Notes. 2018 Jun 7;11:360. doi: 10.1186/s13104-018-3457-8 (PMC5992677; doi:10.1186/s13104-018-3457-8)
Supplement: Supplementary file 1 — Additional file 1. Overview of qPCR protocols for detection of stx, eae, other E. coli pathogenic markers, serotyping genes and indicator markers. [file 13104_2018_3457_MOESM1_ESM.docx]

**Additional file 1.** Overview of qPCR protocols for detection of *stx*, *eae*, other *E. coli* pathogenic markers, serotyping genes and indicator markers

| Pathogenic markers | | | | | | | | | | | Serotyping | | | | Indicator markers | | | | | | | | | | | IAC | Ref. |
| --- | --- | --- | --- | --- | --- | --- | --- | --- | --- | --- | --- | --- | --- | --- | --- | --- | --- | --- | --- | --- | --- | --- | --- | --- | --- | --- | --- |
|  |  |  |  |  |  |  |  |  |  |  |  |  |  |  | Housekeeping genes | | | | | Specific genes | | | | | |  |  |
| *stx_1_* | *stx_2_* | All *stx* | *eae* | *bfpA*  EAF | *aggR CVD432* | *hlyA* | *elt AB* | *esth estp* | *invA-E*  *ipaH* | *astA* | *eae* | *rfb*, *wzx* | *fli* | *Uid A* Z3276* | 16-23S rDNA | *uspA* | *alr* | *mdh* | *wec* (*rfe/rff*) | *phoA* | *Uid A/R* | *gad AB* | *lamB* | *lacZ* | *tuf* |  |  |
| **+** | **+** |  |  |  |  |  |  |  |  |  |  |  |  |  |  |  |  |  |  |  |  |  |  |  |  |  | [1-5] |
|  |  | **+** |  |  |  |  |  |  |  |  |  |  |  |  |  |  |  |  |  |  |  |  |  |  |  |  | [6-12] |
|  |  |  | **+** |  |  |  |  |  |  |  |  |  |  |  |  |  |  |  |  |  |  |  |  |  |  |  | [13-15] |
|  |  |  |  |  |  |  |  |  |  |  |  | **+** |  |  |  |  |  |  |  |  |  |  |  |  |  |  | [16-21] |
|  |  |  | **+** | **+** |  |  |  |  |  |  |  |  |  |  |  |  |  |  |  |  |  |  |  |  |  |  | [22] |
| **+** | **+** |  | **+** |  |  |  |  |  |  |  |  |  |  |  |  |  |  |  |  |  |  |  |  |  |  |  | [23-26] |
| **+** | **+** |  |  |  |  |  | **+** |  |  |  |  |  |  |  |  |  |  |  |  |  |  |  |  |  |  |  | [27] |
| **+** | **+** |  |  |  |  |  |  |  | **+** |  |  |  |  |  |  |  |  |  |  |  |  |  |  |  |  |  | [28] |
| **+** | **+** |  |  |  |  |  |  |  |  |  |  |  | **+** |  |  |  |  |  |  |  |  |  |  |  |  |  | [29] |
| **+** | **+** |  |  |  |  |  |  |  |  |  |  |  |  | **+*** |  |  |  |  |  |  |  |  |  |  |  |  | [30] |
| **+** | **+** |  | **+** |  |  | **+** |  |  |  |  |  |  |  |  |  |  |  |  |  |  |  |  |  |  |  |  | [18, 31] |
| **+** | **+** |  | **+** |  |  |  |  |  |  |  |  | **+** |  |  |  |  |  |  |  |  |  |  |  |  |  |  | [32] |
| **+** | **+** |  |  |  |  |  | **+** | **+** |  |  |  |  |  |  |  |  |  |  |  |  |  |  |  |  |  |  | [33] |
| **+** | **+** |  |  |  |  |  | **+** | **+** | **+** |  |  |  |  |  |  |  |  |  |  |  |  |  |  |  |  |  | [22] |
| **+** | **+** |  | **+** | **+** |  |  | **+** | **+** |  |  |  |  |  |  |  |  |  |  |  |  |  |  |  |  |  |  | [34] |
| **+** | **+** |  |  |  |  | **+** |  |  |  |  | **+** |  |  |  |  |  |  |  |  |  |  |  |  |  |  |  | [35] |
| **+** | **+** |  |  |  |  |  |  |  |  |  | **+** |  | **+** |  |  |  |  |  |  |  |  |  |  |  |  |  | [36] |
| **+** | **+** |  | **+** |  |  |  |  |  |  |  |  | **+** | **+** |  |  |  |  |  |  |  |  |  |  |  |  |  | [37] |
| **+** | **+** |  | **+** |  |  | **+** |  |  |  |  |  |  |  | **+*** |  |  |  |  |  |  |  |  |  |  |  |  | [38] |
| **+** | **+** |  | **+** | **++** | **++** |  | **+** | **++** | **+** | **+** |  |  |  |  |  |  |  |  |  |  |  |  |  |  |  |  | [39] |
|  |  | **+** |  |  |  | **+** |  |  |  |  | **+** |  |  |  |  |  |  |  |  |  |  |  |  |  |  |  | [40] |
|  |  | **+** |  |  |  | **+** |  |  |  |  |  | **+** |  |  |  |  |  |  |  |  |  |  |  |  |  |  | [41] |
|  |  | **+** |  |  |  |  |  |  |  |  |  | **+** | **+** |  |  |  |  |  |  |  |  |  |  |  |  |  | [42] |
|  |  | **+** |  |  |  | **+** |  |  | **+** |  | **+** |  |  |  |  |  |  |  |  |  |  |  |  |  |  |  | [43] |
|  |  | **+** | **+** |  | **+** |  | **+** | **+** | **+** |  |  |  |  |  |  |  |  |  |  |  |  |  |  |  |  |  | [44] |
|  |  |  |  |  |  |  |  |  |  |  |  |  |  |  | **+** |  |  |  |  |  |  |  |  |  |  |  | [45-49] |
|  |  |  |  |  |  |  |  |  |  |  |  |  |  |  |  | **+** |  |  |  |  |  |  |  |  |  |  | [50] |
|  |  |  |  |  |  |  |  |  |  |  |  |  |  |  |  |  | **+** |  |  |  |  |  |  |  |  |  | [51, 52] |
|  |  |  |  |  |  |  |  |  |  |  |  |  |  |  |  |  |  | **+** |  |  |  |  |  |  |  |  | [53] |
|  |  |  |  |  |  |  |  |  |  |  |  |  |  |  |  |  |  |  |  |  | **+** |  |  |  |  |  | [54, 55] |
|  |  |  |  |  |  |  |  |  |  |  |  |  |  |  |  |  |  |  |  |  |  | **+** |  |  |  |  | [54] |
|  |  |  |  |  |  |  |  |  |  |  |  |  |  |  |  |  |  |  |  |  |  |  |  |  | **+** |  | [56-58] |
|  |  |  |  |  |  |  |  |  |  |  |  |  |  |  | **+** |  |  |  |  |  | **+** |  |  |  |  |  | [59] |
|  |  |  |  |  |  |  |  |  |  |  |  |  |  |  |  |  |  |  |  |  | **+** |  | **+** | **+** |  |  | [60] |
|  |  |  |  |  |  |  |  |  |  |  |  |  |  |  |  |  |  |  | **+** |  |  |  |  |  |  |  | [61] |
|  |  |  |  |  |  |  | **+** | **+** |  |  |  |  |  |  |  | **+** |  |  |  |  |  |  |  |  |  |  | [62] |
| **+** | **+** |  |  |  |  |  |  |  |  |  |  |  |  |  |  |  |  |  |  |  |  | **+** |  |  |  |  | [63] |
|  |  |  |  |  |  |  |  |  |  |  |  | **+** |  |  |  |  |  |  |  |  | **+** |  |  |  |  |  | [64] |
| **+** | **+** |  |  |  |  |  |  |  |  |  |  | **+** |  |  |  |  |  |  |  |  | **+** |  |  |  |  |  | [65] |
| **+** | **+** |  | **+** |  |  |  |  |  |  |  |  |  |  |  | **+** |  |  |  |  |  |  |  |  |  |  |  | [66] |
| **+** | **+** |  | **+** |  |  |  |  |  |  |  |  | **+** |  |  | **+** |  |  |  |  |  |  |  |  |  |  |  | [67] |
| **+** | **+** |  | **+** |  |  |  | **+** | **+** | **+** |  |  |  |  |  | **+** |  |  |  |  |  |  |  |  |  |  |  | [68] |
| **+** | **+** |  | **+** | **+** |  |  | **+** | **+** | **+** |  |  |  |  |  | **+** |  |  |  |  |  |  |  |  |  |  |  | [69] |
| **+** |  |  |  |  |  |  |  |  | **+** |  |  |  |  |  |  |  |  |  |  |  | **+** |  |  |  |  |  | [70] |
|  | **+** |  |  |  |  | **+** |  |  |  |  |  | **+** | **+** |  | **+** |  |  |  |  |  |  |  |  |  |  |  | [66] |
|  |  | **+** | **+** |  |  |  | **+** | **+** |  |  |  |  |  |  |  |  |  |  |  | **+** |  |  |  |  |  |  | [71] |
| **+** | **+** |  |  |  |  |  |  |  |  |  |  |  |  | **+** |  |  |  |  |  |  |  |  |  |  |  | **+** | [72] |
|  |  |  |  |  |  |  |  |  |  |  |  | **+** |  |  |  |  |  |  |  |  |  |  |  |  |  | **+** | [73] |
| **+** | **+** |  |  |  |  |  |  |  |  |  |  |  |  |  |  |  |  |  |  |  |  |  |  |  |  | **+** | [74, 75] |
| **+** | **+** | **+** | **+** |  |  |  |  |  |  |  |  |  |  |  |  |  |  |  | **+** |  |  |  |  |  |  |  | This study |

IAC: Internal Amplification Control; *stx*: shiga toxin; *eae*: *E. coli* attaching and effacing gene coding for intimin, localized on the Locus of Enterocyte Effacement (LEE) and is required for producing the attaching-and-effacing (A/E) phenotype; *bfpA*: bundle-forming pilus gene encoding for the structural protein BFP located on the EAF 50 to 70-Mda virulence plasmid; EAF: EPEC adherence factor virulence plasmid, responsible for the Localised Adherence (LA) phenotype displayed by EPEC strains; aggR: transcriptional activator of AAF/I and AAF/II; CVD432: part of the virulence plasmid for EAggEC; *hlyA*: hemolysin localized on the 60Mda EHEC plasmid; *eltAB*: coding for heat labile enterotoxin (Enterotoxin LT); *esth* & est*p*: coding for heat stable enterotoxin (Enterotoxin ST); *invA*-*E*: genes involved with *Salmonella* invasivness; *ipaH*: gene involved with the enteroinvasive mechanism of EIEC and *Shigella* (sequences present at multiple sites on both the large invasive plasmid and the chromosome); *astA* codes for EAST1, an enterotoxin widely distributed in diarrheagenic *E. coli*; rfb: operon involved with the synthesis of the O-antigen of gram-negative bacteria (comprising the *wzx* O-antigen flippase & *wzy* O-antigen polymerase genes); *fli*: coding for flagelin responsible for the H-antigen; *Z3276*: putative fimbriae protein; *uidA*, β-D-glucuronidase structural gene; *uidA**, SNP specific to *E. coli* O157:H7; *uidR*, upstream regulatory region of β-D-glucuronidase structural gene; 16-23S rDNA: genes coding for the ribosomal RNA 16S and 23S; *uspA*: universal stress protein; *alr*: alanine racemase (provides D-alanine to the peptidoglycan); *mdh*: malic acid dehydrogenase; *wec*F (rff): Involved with the synthesis of the Enterobacterial common antigen (ECA) ; *phoA* alkaline phosphatase; *gadA/B*, glutamate decarboxylase; *lamB*: gene coding for the phage lambda receptor protein, maltose high-affinity uptake system; *lacZ*: β -D-galactosidase structural gene; *tuf*, chromosomal, elongation factor EF-Tu; IAC: Internal Amplification Control;

References

1. Pollard DR, Johnson WM, Lior H, Tyler SD, Rozee KR: **Rapid and specific detection of verotoxin genes in Escherichia coli by the polymerase chain reaction [published erratum appears in J. Clin. Microbiol. 1990 Jun;28(6):1491]**. *Journal of clinical microbiology* 1990, **28**(3):540-545.

2. Olsvik O, Rimstad E, Hornes E, Strockbine N, Wasteson Y, Lund A, Wachsmuth K: **A nested PCR followed by magnetic separation of amplified fragments for detection of Escherichia coli Shiga-like toxin genes**. *Molecular and Cellular Probes* 1991, **5**(6):429-435.

3. Gannon VP, King RK, Kim JY, Thomas EJ: **Rapid and sensitive method for detection of Shiga-like toxin-producing Escherichia coli in ground beef using the polymerase chain reaction**. *Appl Environ Microbiol* 1992, **58**(12):3809-3815.

4. Bellin T, Pulz M, Matussek A, Hempen HG, Gunzer F: **Rapid detection of enterohemorrhagic escherichia coli by real-time PCR with fluorescent hybridization probes**. *Journal of clinical microbiology* 2001, **39**(1):370-374.

5. Lefterova MI, Slater KA, Budvytiene I, Dadone PA, Banaei N: **A Sensitive Multiplex, Real-Time PCR Assay for Prospective Detection of Shiga Toxin-Producing Escherichia coli from Stool Samples Reveals Similar Incidences but Variable Severities of Non-O157 and O157 Infections in Northern California**. *Journal of clinical microbiology* 2013, **51**(9):3000-3005.

6. Fach P, Perelle S, Dilasser F, Grout J: **Comparison between a PCR-ELISA test and the vero cell assay for detecting Shiga toxin-producing Escherichia coli in dairy products and characterization of virulence traits of the isolated strains**. *Journal of Applied Microbiology* 2001, **90**(5):809-818.

7. Karch H, Meyer T: **Single primer pair for amplifying segments of distinct Shiga-like-toxin genes by polymerase chain reaction**. *Journal of clinical microbiology* 1989, **27**(12):2751-2757.

8. Lin Z, Kurazono H, Yamasaki S, Takeda Y: **Detection of Various Variant Verotoxin Genes in *Escherichia coli* by Polymerase Chain Reaction**. *Microbiol Immunol* 1993, **37**(7):543-548.

9. Paton AW, Paton JC, Goldwater PN, Manning PA: **Direct detection of Escherichia coli Shiga-like toxin genes in primary fecal cultures by polymerase chain reaction**. *Journal of clinical microbiology* 1993, **31**(11):3063-3067.

10. Read SC, Clarke RC, Martin A, De Grandis SA, Hii J, McEwen S, Gyles CL: **Polymerase chain reaction for detection of verocytotoxigenic Escherichia coli isolated from animal and food sources**. *Molecular and Cellular Probes* 1992, **6**(2):153-161.

11. Yamasaki S, Lin Z, Shirai H, Terai A, Oku Y, Ito H, Ohmura M, Karasawa T, Tsukamoto T, Kurazono H *et al*: **Typing of verotoxins by DNA colony hybridization with poly- and oligonucleotide probes, a bead-enzyme-linked immunosorbent assay, and polymerase chain reaction**. *Microbiol Immunol* 1996, **40**(5):345-352.

12. Ge B, Zhao S, Hall R, Meng J: **A PCR-ELISA for detecting Shiga toxin-producing Escherichia coli**. *Microbes Infect* 2002, **4**(3):285-290.

13. Meng J, Zhao S, Doyle MP, Mitchell SE, Kresovich S: **Polymerase chain reaction for detecting Escherichia coli O157: H7**. *International journal of food microbiology* 1996, **32**(1-2):103-113.

14. Batchelor M, Knutton S, Caprioli A, Huter V, Zanial M, Dougan G, Frankel G: **Development of a Universal Intimin Antiserum and PCR Primers**. *Journal of clinical microbiology* 1999, **37**(12):3822-3827.

15. Karch H, Bohm H, Schmidt H, Gunzer F, Aleksic S, Heesemann J: **Clonal structure and pathogenicity of Shiga-like toxin-producing, sorbitol-fermenting Escherichia coli O157:H-**. *Journal of clinical microbiology* 1993, **31**(5):1200-1205.

16. Desmarchelier PM, Bilge SS, Fegan N, Mills L, Vary JC, Jr, Tarr PI: **A PCR specific for Escherichia coli O157 based on the rfb locus encoding O157 lipopolysaccharide**. *Journal of clinical microbiology* 1998, **36**:1801-1804.

17. Maurer JJ, Schmidt D, Petrosko P, Sanchez S, Bolton L, Lee MD: **Development of primers to O-antigen biosynthesis genes for specific detection of escherichia coli O157 by PCR [In Process Citation]**. *Appl Environ Microbiol* 1999, **65**(7):2954-2960.

18. Paton AW, Paton JC: **Detection and characterization of Shiga toxigenic Escherichia coli by using multiplex PCR assays for stx1, stx2, eaeA, enterohemorrhagic E. coli hlyA, rfbO111, and rfbO157**. *Journal of clinical microbiology* 1998, **36**:598-602.

19. Paton AW, Paton JC: **Direct Detection of Shiga Toxigenic Escherichia coli Strains Belonging to Serogroups O111, O157, and O113 by Multiplex PCR**. *Journal of clinical microbiology* 1999, **37**(10):3362-3365.

20. Abdulmawjood A, Bulte M, Cook N, Roth S, Schonenbrucher H, Hoorfar J: **Toward an international standard for PCR-based detection of Escherichia coli O157: Part 1. Assay development and multi-center validation**. *Journal of Microbiological Methods* 2003, **55**(3):775-786.

21. Sánchez S, Llorente MT, Echeita MA, Herrera-León S: **Development of Three Multiplex PCR Assays Targeting the 21 Most Clinically Relevant Serogroups Associated with Shiga Toxin-Producing E. coli Infection in Humans**. *PLoS One* 2015, **10**(1):e0117660.

22. Aranda KRS, Fagundes-Neto U, Scaletsky ICA: **Evaluation of Multiplex PCRs for Diagnosis of Infection with Diarrheagenic Escherichia coli and Shigella spp**. *Journal of clinical microbiology* 2004, **42**(12):5849-5853.

23. China B, Pirson V, Mainil J: **Typing of bovine attaching and effacing Escherichia coli by multiplex in vitro amplification of virulence-associated genes**. *Appl Environ Microbiol* 1996, **62**(9):3462-3465.

24. Heuvelink AE, Van-De-Kar N-CA, Meis J-FG, Monnens L-AH, Melchers W-JG: **Characterization of verocytotoxin-producing Escherichia coli O157 isolates from patients with haemolytic uraemic syndrome in western Europe**. *Epidemiol Infect* 1995, **115**:1-14.

25. Pass MA, Odedra R, Batt RM: **Multiplex PCRs for Identification of Escherichia coli Virulence Genes**. *Journal of clinical microbiology* 2000, **38**(5):2001-2004.

26. Meng J, Zhao S, Doyle MP, Mitchell SE, Kresovich S: **A multiplex PCR for identifying Shiga-like toxin-producing Escherichia coli O157:H7**. *Letters in applied microbiology* 1997, **24**(3):172-176.

27. Lang AL, Tsai YL, Mayer CL, Patton KC, Palmer CJ: **Multiplex PCR for detection of the heat-labile toxin gene and shiga-like toxin I and II genes in Escherichia coli isolated from natural waters**. *Appl Environ Microbiol* 1994, **60**(9):3145-3149.

28. Chen J, Griffiths MW: **Detection of Salmonella and simultaneous detection of Salmonella and Shiga-like toxin-producing Escherichia coli using the magnetic capture hybridization polymerase chain reaction**. *Letters in applied microbiology* 2001, **32**(1):7-11.

29. Radu S, Ling OW, Rusul G, Karim MI, Nishibuchi M: **Detection of Escherichia coli O157:H7 by multiplex PCR and their characterization by plasmid profiling, antimicrobial resistance, RAPD and PFGE analyses**. *Journal of Microbiology Methods* 2001, **46**(2):131-139.

30. Cebula TA, Payne WL, Feng P: **Simultaneous identification of strains of Escherichia coli serotype O157:H7 and their Shiga-like toxin type by mismatch amplification mutation assay-multiplex PCR [published erratum appears in J Clin Microbiol 1995 Apr;33(4):1048]**. *JClinMicrobiol* 1995, **33**(1):248-250.

31. Call DR, Brockman FJ, Chandler DP: **Detecting and genotyping Escherichia coli O157:H7 using multiplexed PCR and nucleic acid microarrays**. *International journal of food microbiology* 2001, **67**(1-2):71-80.

32. Osek J: **Rapid and specific identification of Shiga toxin-producing Escherichia coli in faeces by multiplex PCR**. *Letters in applied microbiology* 2002, **34**(4):304-310.

33. Tsen HY, Jian LZ: **Development and use of a multiplex PCR system for the rapid screening of heat labile toxin I, heat stable toxin II and shiga-like toxin I and II genes of Escherichia coli in water**. *Journal of Applied Microbiology* 1998, **84**(4):585-592.

34. Vidal R, Vidal M, Lagos R, Levine M, Prado V: **Multiplex PCR for diagnosis of enteric infections associated with diarrheagenic Escherichia coli**. *Journal of clinical microbiology* 2004, **42**(4):1787-1789.

35. Fagan PK, Hornitzky MA, Bettelheim KA, Djordjevic SP: **Detection of shiga-like toxin (stx1 and stx2), intimin (eaeA), and enterohemorrhagic Escherichia coli (EHEC) hemolysin (EHEC hlyA) genes in animal feces by multiplex PCR**. *Appl Environ Microbiol* 1999, **65**(2):868-872.

36. Gannon VP, D'Souza S, Graham T, King RK, Rahn K, Read: **Use of the flagellar H7 gene as a target in multiplex PCR assays and improved specificity in identification of enterohemorrhagic Escherichia coli strains**. *Journal of clinical microbiology* 1997, **35**(3):656-662.

37. Hu Y, Zhang Q, Meitzler JC: **Rapid and sensitive detection of Escherichia coli O157:H7 in bovine faeces by a multiplex PCR**. *Journal of Applied Microbiology* 1999, **87**(6):867-876.

38. Feng P, Monday SR: **Multiplex PCR for detection of trait and virulence factors in enterohemorrhagic escherichia coli serotypes [In Process Citation]**. *Mol Cell Probes* 2000, **14**(6):333-337.

39. Kimata K, Shima T, Shimizu M, Tanaka D, Isobe J, Gyobu Y, Watahiki M, Nagai Y: **Rapid categorization of pathogenic Escherichia coli by multiplex PCR**. *Microbiol Immunol* 2005, **49**(6):485-492.

40. Fratamico PM, Bhaduri S, Buchanan RL: **Studies on *Escherichia coli* serotype 0157:H7 strains containing a 60-MDa plasmid and on 60-MDa plasmid-cured derivates**. *J Med Microbiol* 1993, **39**(5):371-381.

41. Osek J, Dacko J: **Development of a PCR-based method for specific identification of genotypic markers of shiga toxin-producing Escherichia coli strains**. *Zoonoses Public Hlth* 2001, **48**(10):771-778.

42. Nagano I, Kunishima M, Itoh Y, Wu Z, Takahashi Y: **Detection of verotoxin-producing Escherichia coli O157:H7 by multiplex polymerase chain reaction**. *Microbiol Immunol* 1998, **42**(5):371-376.

43. Fratamico P, Strobaugh TP: **Simultaneous detection of Salmonella spp and Escherichia coli O157:H7 by multiplex PCR**. *J Ind Microbiol Biot* 1998, **21**:92-98.

44. Toma C, Lu Y, Higa N, Nakasone N, Chinen I, Baschkier A, Rivas M, Iwanaga M: **Multiplex PCR Assay for Identification of Human Diarrheagenic Escherichia coli**. *Journal of clinical microbiology* 2003, **41**(6):2669.

45. Carroll NM, Jaeger EE, Choudhury S, Dunlop AA, Matheson MM, Adamson P, Okhravi N, Lightman S: **Detection of and Discrimination between Gram-Positive and Gram-Negative Bacteria in Intraocular Samples by Using Nested PCR**. *Journal of clinical microbiology* 2000, **38**(5):1753-1757.

46. Klausegger A, Hell M, Berger A, Zinober K, Baier S, Jones N, Sperl W, Kofler B: **Gram type-specific broad-range PCR amplification for rapid detection of 62 pathogenic bacteria [published erratum appears in J Clin Microbiol 1999 May;37(5):1660]**. *Journal of clinical microbiology* 1999, **37**(2):464-466.

47. Mittelman MW, Habash M, Lacroix J-M, Khoury AE, Krajden: **Rapid detection of Enterobacteriaceae in urine by fluorescent 16S rRNA in situ hybridization on membrane filters**. *Journal of Microbiological Methods* 1997, **30**(2):153-160.

48. Greisen K, Loeffelholz M, Purohit A, Leong D: **PCR primers and probes for the 16S rRNA gene of most species of pathogenic bacteria, including bacteria found in cerebrospinal fluid**. *JClinMicrobiol* 1994, **32**(2):335-351.

49. Nakano S, Kobayashi T, Funabiki K, Matsumura A, Nagao Y, Yamada T: **Development of a PCR assay for detection of Enterobacteriaceae in foods**. *Journal of food protection* 2003, **66**(10):1798-1804.

50. Chen J, Griffiths MW: **PCR differentiation of Escherichia coli from other Gram-negative bacteria using primers derived from the nucleotide sequences flanking the gene encoding the universal stress protein**. *Letters in applied microbiology* 1998, **27**(6):369-371.

51. Daly P, Collier T, Doyle S: **PCR-ELISA detection of Escherichia coli in milk**. *Letters in applied microbiology* 2002, **34**(3):222-226.

52. Yokoigawa K, Inoue K, Okubo Y, Kawai H: **Primers for amplifying an alanine racemase gene fragment to detect E. coli strains in foods**. *Journal of food science* 1999, **64**(4):571-574.

53. Hsu SC, Tsen HY: **PCR primers designed from malic acid dehydrogenase gene and their use for detection of Escherichia coli in water and milk samples**. *IntJ Food Microbiol* 2001, **64**(1-2):1-11.

54. McDaniels AE, Rice EW, Reyes AL, Johnson CH, Haugland, RA, Stelma GN, Jr.: **Confirmational identification of Escherichia coli, a comparison of genotypic and phenotypic assays for glutamate decarboxylase and beta-D-glucuronidase**. *Appl Environ Microbiol* 1996, **62**(9):3350-3354.

55. Bej AK, Dicesare JL, Haff L, Atlas RM: **Detection of Escherichia coli and Shigella spp. in water by using the polymerase chain reaction and gene probes for uid**. *Appl Environ Microbiol* 1991, **57**(4):1013-1017.

56. Ke D, Picard FJ, Martineau F, Menard C, Roy PH, Ouellette M, Bergeron MG: **Development of a PCR assay for rapid detection of enterococci [In Process Citation]**. *Journal of clinical microbiology* 1999, **37**(11):3497-3503.

57. Maheux AF, Bérubé È, Boudreau DK, Cantin P, Boissinot M, Bissonnette L, Rodrigue L, Bergeron MG: **Ability of three DNA-based assays to identify presumptive Escherichia coli colonies isolated from water by the culture-based mFC agar method**. *Water Research* 2011, **45**(8):2638-2646.

58. Maheux AF, Picard FJ, Boissinot M, Bissonnette L, Paradis S, Bergeron MG: **Analytical comparison of nine PCR primer sets designed to detect the presence of Escherichia coli/Shigella in water samples**. *Water Research* 2009, **43**(12):3019-3028.

59. Frahm E, Obst U: **Application of the fluorogenic probe technique (TaqMan PCR) to the detection of Enterococcus spp. and Escherichia coli in water samples**. *Journal of Microbiological Methods* 2003, **52**(1):123-131.

60. Bej AK, Mahbubani MH, Dicesare JL, Atlas RM: **Polymerase chain reaction-gene probe detection of microorganisms by using filter-concentrated samples**. *Appl Environ Microbiol* 1991, **57**(12):3529-3534.

61. Bayardelle P, Zafarullah M: **Development of oligonucleotide primers for the specific PCR-based detection of the most frequent Enterobacteriaceae species DNA using wec gene templates**. *Canadian journal of microbiology* 2002, **48**(2):113-122.

62. Osek J: **Multiplex polymerase chain reaction assay for identification of enterotoxigenic Escherichia coli strains**. *J Vet Diagn Invest* 2001, **13**(4):308-311.

63. Grant MA, Weagant SD, Feng P: **Glutamate Decarboxylase Genes as a Prescreening Marker for Detection of Pathogenic Escherichia coli Groups**. *Appl Environ Microbiol* 2001, **67**(7):3110-3114.

64. Anklam KS, Kanankege KST, Gonzale TK, Kaspar CW, Dopfer D: **Rapid and Reliable Detection of Shiga Toxin–Producing Escherichia coli by Real-Time Multiplex PCR**. *Journal of food protection* 2012, **75**(4):643-650.

65. Heijnen L, Medema G: **Quantitative detection of E. coli, E. coli O157 and other shiga toxin producing E. coli in water samples using a culture method combined with real-time PCR**. *Journal of water and health* 2006, **4**(4):487-498.

66. Wang G, Clark CG, Rodgers FG: **Detection in Escherichia coli of the genes encoding the major virulence factors, the genes defining the O157:H7 serotype, and components of the type 2 Shiga toxin family by multiplex PCR**. *Journal of clinical microbiology* 2002, **40**(10):3613-3619.

67. Monday SR, Beisaw A, Feng PCH: **Identification of Shiga toxigenic Escherichia coli seropathotypes A and B by multiplex PCR**. *Molecular and Cellular Probes* 2007, **21**(4):308-311.

68. Persson S, Olsen KE, Scheutz F, Krogfelt KA, Gerner-Smidt P: **A method for fast and simple detection of major diarrhoeagenic Escherichia coli in the routine diagnostic laboratory**. *Clinical Microbiology and Infection* 2007, **13**(5):516-524.

69. Brandal LT, Lindstedt BA, Aas L, Stavnes TL, Lassen J, Kapperud G: **Octaplex PCR and fluorescence-based capillary electrophoresis for identification of human diarrheagenic Escherichia coli and Shigella spp**. *Journal of Microbiological Methods* 2007, **68**(2):331-341.

70. Riyaz-Ul-Hassan S, Syed S, Johri S, Verma V, Qazi GN: **Application of a multiplex PCR assay for the detection of Shigella, Escherichia coli and Shiga toxin-producing Esch. coli in milk**. *Journal of Dairy Research* 2009, **76**(2):188-194.

71. Kong RYC, So CL, Law WF, Wu RSS: **A Sensitive and Versatile Multiplex PCR System for the Rapid Detection of Enterotoxigenic (ETEC), Enterohaemorrhagic (EHEC) and Enteropathogenic (EPEC) Strains of Escherichia coli**. *Marine pollution bulletin* 1999, **38**(12):1207-1215.

72. Li B, Liu H, Wang W: **Multiplex real-time PCR assay for detection of Escherichia coli O157:H7 and screening for non-O157 Shiga toxin-producing E. coli**. *Bmc Microbiol* 2017, **17**(1):215.

73. Singh P, Mustapha A: **Multiplex real-time PCR assays for detection of eight Shiga toxin-producing Escherichia coli in food samples by melting curve analysis**. *International journal of food microbiology* 2015, **215**:101-108.

74. Salinas-Ibáñez ÁG, Lucero-Estrada C, Chialva C, Zárate JM, Juri-Ayub M, Escudero ME: **Design of an internal amplification control for a duplex PCR used in the detection of Shiga toxin producing Escherichia coli in pediatric feces**. *Molecular and Cellular Probes* 2015, **29**(6):351-357.

75. Brusa V, Galli L, Linares LH, Ortega EE, Lirón JP, Leotta GA: **Development and validation of two SYBR green PCR assays and a multiplex real-time PCR for the detection of Shiga toxin-producing Escherichia coli in meat**. *Journal of Microbiological Methods* 2015, **119**:10-17.
