# Supplementary material for: Multiplex PCR for the simultaneous detection of the Enterobacterial gene wecA, the Shiga Toxin genes (stx1 and stx2) and the Intimin gene (eae)
Source: BMC Res Notes. 2018 Jun 7;11:360. doi: 10.1186/s13104-018-3457-8 (PMC5992677; doi:10.1186/s13104-018-3457-8)
Supplement: Supplementary file 2 — Additional file 2. List of strains with triplex PCR results. [file 13104_2018_3457_MOESM2_ESM.docx]

**Additional file 2.** List of strains with triplex PCR results

| Numbering used in Figures | Species | Strain | *wecA (rfe)* | *eae* | *stx* |
| --- | --- | --- | --- | --- | --- |
| 1 | EHEC O128:H? | BE97-2317 | + | - | + |
| 2 | EHEC O113:H21 | BE96-2409 | + | - | + |
| 3 | EHEC O157:H7 | 3070/00 | + | + | + |
| 4 | EHEC O157:H7 | 4288/84 | + | + | + |
| 5 | EHEC O?:H? | 385/97 | + | + | + |
| 6 | *S. dysenteriae* serotype 1 | 605/94 | + | - | + |
| 7 | *S. dysenteriae* serotype 1 | 53/95 | + | - | + |
| 8 | *S. dysenteriae* serotype 1 | 3022/00 | + | - | + |
| 9 | EHEC O157:H7 | 1480/96 | + | + | + |
| 10 | EHEC O157:H- | 1113/93 | + | + | + |
| 11 | EHEC O157:H? | 956/98 | + | + | + |
| 12 | EHEC O157:H7 | 2610/00 | + | + | + |
| 13 | EHEC O157:H7 | 2889/00 | + | + | + |
| 14 | EHEC O157:H7 | 3002/00 | + | + | + |
| 15 | EHEC O157:H7 | 3005/00 | + | + | - |
| 16 | EHEC O157:H7 | ATCC 43888 | + | + | - |
| 17 | EPEC O26:K60 | 573/96 | + | + | - |
| 18 | EPEC O127:K63 | 2320/96 | + | + | - |
| 19 | EPEC O26:K60 |  | + | + | - |
| 20 | EPEC O55:K59 |  | + | + | - |
| 21 | EPEC O128:K67 |  | + | + | - |
| 22 | EPEC O119:K69 | 557/96 | + | + | - |
| 23 | *Escherichia coli* | ATCC 25922 | + | - | - |
