# Supplementary material for: Multiplex PCR for the simultaneous detection of the Enterobacterial gene wecA, the Shiga Toxin genes (stx1 and stx2) and the Intimin gene (eae)
Source: BMC Res Notes. 2018 Jun 7;11:360. doi: 10.1186/s13104-018-3457-8 (PMC5992677; doi:10.1186/s13104-018-3457-8)
Supplement: Supplementary file 3 — Additional file 3. Comparison of selected primers aiming at the detection of all variants of stxA gene; arrows are indicating the direction of the primers and complementary sequences are shown for the reverse primers. The conserved bases are shaded in black while the variable positions are unshaded. [file 13104_2018_3457_MOESM3_ESM.docx]

Amplicon size (bp)

227-224

900

323

220

215-212

521-518

523-520

526-523

523

[1]: GAGCGAAATAATTTATATGTG------//------------------------------------------------------------------

[2]: ----GAACGAAATAATTTATATGT ---//------------------------------------------------------------------
[3]: ----GAGCGAAATAATTTATATGT ---//---- GGCAAATACAGAGGGGATTTCG------------------------------------

[4]: ------------------------------------------GCAGATACAGAGAGAATTTCGT //-- ATACTGAATTGCCA-TCATCAG-

[5]: ----------------------------------------------ATACAGAGRGRATTTCGT //-- ATACTGAATTGYCA-TCATCA--

[6]: ----GAGCGAAATAATTTATATGTG ---------------//------------------------- ATACTGAATTGCCA-TCATCA--

[7]: ----GAACGAAATAATTTATATGTG ---------------//-------------------------- TACTGAATTGCCA-TCATCAGG

UstxU1&L1: TRTTGARCRAAATAATTTATATGT --------------- //------------------------- ATACTGAATTGYCA-TCATCAK-

UstxU3&L3: AATGGAACGGAATAACTTATATGT -------------- //-------------------------- ATCCTTAATTGCCACTCAACC--

**Additional file 3.** Comparison of selected primers aiming at the detection of all variants of *stxA* gene; arrows are indicating the direction of the primers and complementary sequences are shown for the reverse primers. The conserved bases are shaded in black while the variable positions are unshaded.

References

1. Karch H, Meyer T: **Single primer pair for amplifying segments of distinct Shiga-like-toxin genes by polymerase chain reaction**. *Journal of clinical microbiology* 1989, **27**(12):2751-2757.

2. Lin Z, Kurazono H, Yamasaki S, Takeda Y: **Detection of Various Variant Verotoxin Genes in *Escherichia coli* by Polymerase Chain Reaction**. *Microbiol Immunol* 1993, **37**(7):543-548.

3. Read SC, Clarke RC, Martin A, De Grandis SA, Hii J, McEwen S, Gyles CL: **Polymerase chain reaction for detection of verocytotoxigenic Escherichia coli isolated from animal and food sources**. *Molecular and Cellular Probes* 1992, **6**(2):153-161.

4. Ge B, Zhao S, Hall R, Meng J: **A PCR-ELISA for detecting Shiga toxin-producing Escherichia coli**. *Microbes Infect* 2002, **4**(3):285-290.

5. Paton AW, Paton JC, Goldwater PN, Manning PA: **Direct detection of Escherichia coli Shiga-like toxin genes in primary fecal cultures by polymerase chain reaction**. *Journal of clinical microbiology* 1993, **31**(11):3063-3067.

6. Yamasaki S, Lin Z, Shirai H, Terai A, Oku Y, Ito H, Ohmura M, Karasawa T, Tsukamoto T, Kurazono H *et al*: **Typing of verotoxins by DNA colony hybridization with poly- and oligonucleotide probes, a bead-enzyme-linked immunosorbent assay, and polymerase chain reaction**. *Microbiol Immunol* 1996, **40**(5):345-352.

7. Kong RYC, So CL, Law WF, Wu RSS: **A Sensitive and Versatile Multiplex PCR System for the Rapid Detection of Enterotoxigenic (ETEC), Enterohaemorrhagic (EHEC) and Enteropathogenic (EPEC) Strains of Escherichia coli**. *Marine pollution bulletin* 1999, **38**(12):1207-1215.
