# Supplementary material for: Multiplex PCR for the simultaneous detection of the Enterobacterial gene wecA, the Shiga Toxin genes (stx1 and stx2) and the Intimin gene (eae)
Source: BMC Res Notes. 2018 Jun 7;11:360. doi: 10.1186/s13104-018-3457-8 (PMC5992677; doi:10.1186/s13104-018-3457-8)
Supplement: Supplementary file 4 — Additional file 4. PCR and BsrI digestion. Lane A, DNA size ladder; Lane 1 to 28, stx positive strains as listed in Additional file 2, odd numbers are the undigested stx products, even numbers are the digestion products. [file 13104_2018_3457_MOESM4_ESM.docx]

(62)

526-523 *stx*_1_,_2_

193-200

bp


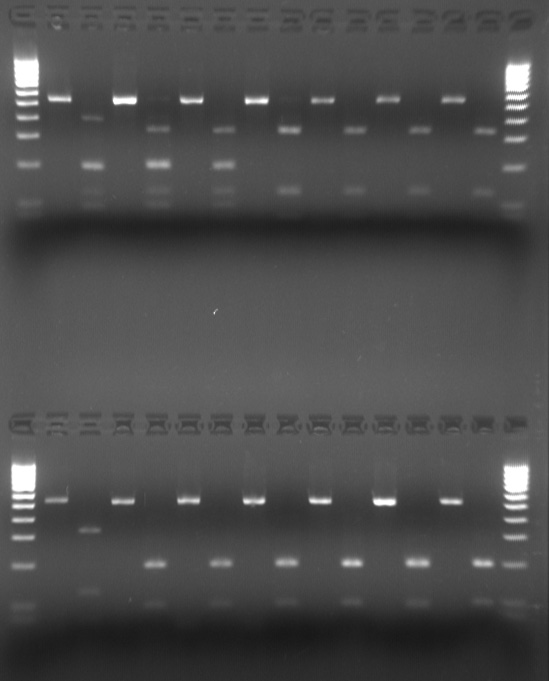


A 1 2 3 4 5 6 7 8 9 10 11 12 13 14 15 16 17 18 19 20 21 22 23 24 25 26 27 28

396

334

91

130

400

1031

500

300

200

100

80


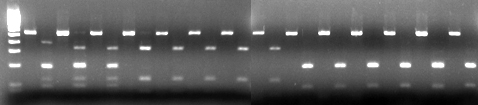


**Additional file 4.** PCR and BsrI digestion. Lane A, DNA size ladder; Lane 1 to 28, stx positive strains as listed in Additional file 2, odd numbers are the undigested stx products, even numbers are the digestion products.
