# Supplementary material for: Multiplex PCR for the simultaneous detection of the Enterobacterial gene wecA, the Shiga Toxin genes (stx1 and stx2) and the Intimin gene (eae)
Source: BMC Res Notes. 2018 Jun 7;11:360. doi: 10.1186/s13104-018-3457-8 (PMC5992677; doi:10.1186/s13104-018-3457-8)
Supplement: Supplementary file 5 — Additional file 5. Subtyping results for stx by restriction enzymatic digestion of the stx amplicon and semi-nested duplex PCR. [file 13104_2018_3457_MOESM5_ESM.docx]

**Additional file 5.** Subtyping results for *stx* by restriction enzymatic digestion of the *stx* amplicon and semi-nested duplex PCR.

| Species | Strain | BsrI digestion | | | | Semi nested duplex PCR |
| --- | --- | --- | --- | --- | --- | --- |
|  |  | *stx*_1c_  (526) | *stx*_1_  (526) | *stx*_2,2c,2d_  (523) | *stx*_2e,2f_  (523) |  |
|  |  | 396 + 130 | 334 + 130 + 62 | 200 & 193 + 91 + 39 | 200 & 193 + 130 |  |
| EHEC O128:H? | BE97-2317 | + | - | + | - | *stx*_1_ *+ stx*_2_ |
| EHEC O113:H21 | BE96-2409 | - | + | + | ? | *stx*_1_ *+ stx*_2_ |
| EHEC O157:H7 | 3070/00 | - | + | + | ? | *stx*_1_ *+ stx*_2_ |
| EHEC O157:H7 | 4288/84 | - | + | - | - | *stx*_1_ |
| EHEC O?:H? | 385/97 | - | + | - | - |  |
| *S. dysenteriae* serotype 1 | 605/94 | - | + | - | - |  |
| *S. dysenteriae* serotype 1 | 53/95 | - | + | - | - |  |
| *S. dysenteriae* serotype 1 | 3022/00 | - | + | - | - |  |
| EHEC O157:H7 | 1480/96 | - | - | + | - | *stx*_2_ |
| EHEC O157:H- | 1113/93 | - | - | + | - |  |
| EHEC O157:H? | 956/98 | - | - | + | - |  |
| EHEC O157:H7 | 2610/00 | - | - | + | - |  |
| EHEC O157:H7 | 2889/00 | - | - | + | - |  |
| EHEC O157:H7, | 3002/00 | - | - | + | - |  |
